# Generative artificial intelligence in diabetes healthcare

**DOI:** 10.1016/j.isci.2025.113051

**Published:** 2025-07-05

**Authors:** Josep Vehi, Omer Mujahid, Aleix Beneyto, Ivan Contreras

**Affiliations:** 1 Modeling and Intelligent Control Engineering Laboratory, Institut d’Informàtica i Aplicacions, Universitat de Girona, 17003 Girona, Spain; 2 Centro de Investigación Biomédica en Red de Diabetes y Enfermedades Metabólicas Asociadas (CIBERDEM), Madrid, Spain

**Keywords:** Health sciences, Diabetology, Artificial intelligence

## Abstract

The rapid advancement of generative artificial intelligence (AI) has been fueled by breakthroughs in large language models and applications across diverse domains, from creative content to scientific discovery. Its strength lies in modeling, simulating, and generating high-fidelity data. In diabetes care, generative AI enables solutions to challenges such as data scarcity, patient variability, and personalization. This article explores key deep generative models, including variational autoencoders, generative adversarial networks, transformers, and diffusion models applied to tabular, time series, image, and text data. These models enable synthetic patient data generation, dataset augmentation, glucose-insulin dynamics simulation, and the development of virtual coaches and digital twins. Despite these advances, challenges persist, including model instability, high data requirements, and output interpretability. This article reviews the current literature and outlines opportunities, limitations, and ethical considerations for the use of generative AI in diabetes healthcare.

## Graphical abstract

**Figure:**
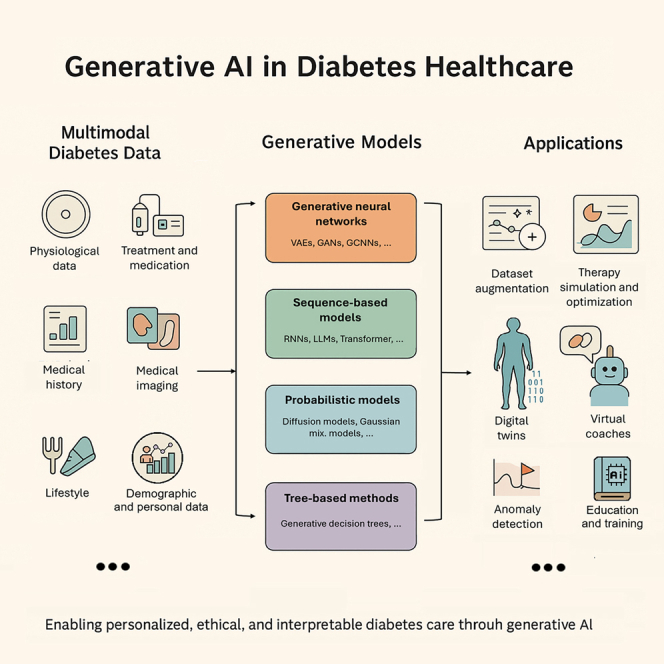

## Introduction

Modern clinical and biomedical research increasingly demands advanced models capable of representing human physiology and health-related processes. Applications such as personalized therapies, adaptive medical devices, and predictive modeling of patient outcomes require simulating complex biological systems and individual patient responses. However, building such models is challenging due to the scarcity of high-quality labeled data, the variability across patient populations, and the dynamic, nonlinear nature of human physiology.

Researchers have developed various modeling techniques, ranging from expert knowledge-based mechanistic models to data-driven empirical models. Mechanistic models rely on first principles, such as compartmental and agent-based modeling, and differential equations. Empirical models fit data, focusing on patterns and relationships without explicit rules. They are useful for complex systems or when data are plentiful but understanding is limited. Mechanistic and data-driven models are vital in system modeling, with data-driven models becoming prominent due to the increasing data availability and advances in machine learning. Among these, generative artificial intelligence (AI) has emerged as a transformative approach, capable of learning complex data distributions and producing realistic synthetic data. Key factors such as architectural advances, powerful hardware, and large datasets have driven its rise, with much of its success attributed to generative deep learning. Notably, large language models (LLMs) and other generative AI applications have exemplified how these advancements can scale across domains, from natural language understanding to multimodal data generation. While recent public attention has largely centered on LLMs, especially in the context of text generation, other forms of generative AI modeling techniques, such as generative adversarial networks (GANs), variational autoencoders (VAEs), and diffusion models, are better suited to tasks involving numerical data, structured time series, and multimodal biomedical signals. These models are particularly valuable in healthcare applications that require modeling complex physiological processes with high quantitative accuracy.

Generative AI is widely used in various domains for generating realistic data, applied in tasks from video generation to drug discovery. Its ability to learn data’s probability distribution makes it useful for approximating dynamic models from physical systems input/output,^1^^,^^2^ sometimes outperforming other techniques.^3^ In healthcare, it is crucial for modeling complex systems, aiding in drug discovery,^4^ personalized medicine,^5^ and developing medical devices with accurate biological simulations.^6^ Generative AI enhances patient care and medical research with the precise modeling of patient data and physiological responses.

## Generative artificial intelligence in diabetes healthcare

The integration of digital health and advancements in diabetes technology has transformed diabetes healthcare, significantly expanding the volume and diversity of available data. Devices such as continuous glucose monitors (CGMs), insulin pumps, and smart pens provide real-time insights into blood glucose levels, insulin dosages, and carbohydrate intake. Smartphone applications complement these tools by tracking meal composition, while clinical data sources contribute lab results, electronic health records (EHRs), and physician notes. This vast array of data also includes behavioral, genomic, and pharmacogenomic studies, along with imaging data such as radiographs and photos of diabetic complications. These datasets are leveraged in training machine learning models and optimizing control systems in diabetes care.

However, modeling diabetes data poses challenges due to the inherent inter- and intra-patient variability.^7^ Traditional computational approaches, such as mechanistic and differential equation models, have limitations in addressing the complexity of the glucose-insulin system and its variability.^8^^,^^9^ Consequently, these methods often produce limited synthetic data and lack generalizability.

To address these limitations, generative AI offers promising solutions by capturing the complexities of diabetes data. Beyond simply generating synthetic data, generative AI contributes to modeling physiological processes, creating personalized treatment plans, and synthesizing diverse datasets. The significant advances in generative AI are largely driven by deep learning techniques. Terminologies such as deep generative models, generative neural networks, and generative deep learning have all been used to refer to the application of deep learning for generative purposes. These approaches share the fundamental goal of capturing the complexities of the data distribution but differ in how they approach learning and generation, with each technique offering its own strengths and trade-offs in terms of performance and application. Understanding these general techniques provides a foundation for exploring how generative AI can be applied specifically to diabetes healthcare.

**Figure 1:**
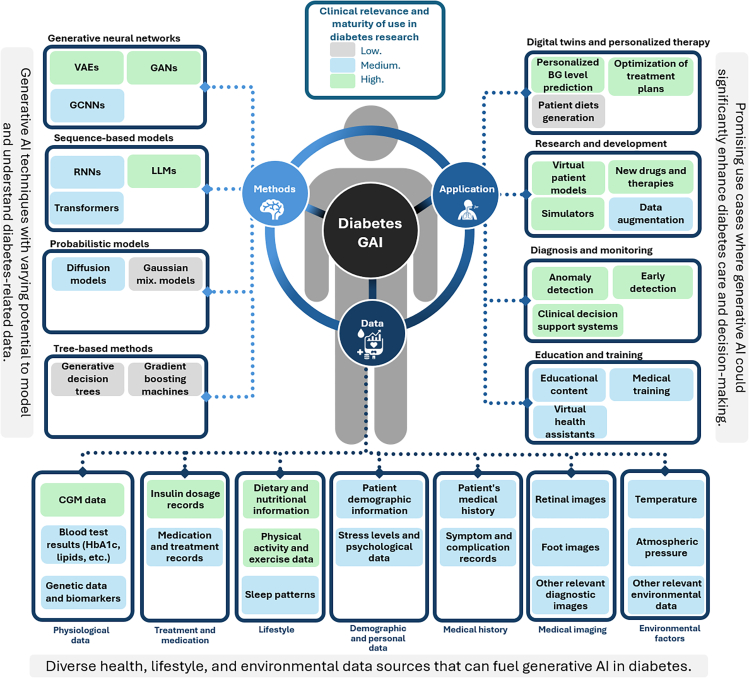
Diabetes generative artificial intelligence: a map of the techniques, applications, and the data that is driving generative artificial intelligence in diabetes

This article explores the application of generative AI in diabetes healthcare. It identifies key modeling approaches and their use cases, supported by insights from the literature. As this article provides a narrative and perspective-driven overview, the aim is not to conduct a formal systematic review, but rather to highlight key developments, models, and trends in the application of generative AI to diabetes care. However, to ground our discussion in relevant evidence, we performed a targeted literature search using Google Scholar and other academic databases. This process yielded 33 key publications spanning major generative model categories—VAEs (9), GANs (15), diffusion models (4), and transformer-based models (5). These works were selected based on their relevance, impact, and alignment with the central themes of this article. An overview of the reviewed literature is presented in Table S1. Figure 1 provides a comprehensive map of the techniques, applications, and data sources driving these innovations, while Figure S1 illustrates the taxonomy of models central to diabetes healthcare. The proposed taxonomy is inspired by prior classifications of generative models in related domains.^10^ These tools guide readers through impactful areas of research and highlight the transformative potential of generative AI in improving diabetes management.

### Density estimation using generative artificial intelligence

Density estimation involves inferring the underlying distribution of a dataset to identify patterns in high-dimensional spaces by estimating the probability density function p(x) from samples. Generative AI enhances this process by approximating complex, multimodal distributions that traditional parametric methods often fail to capture. Through techniques such as likelihood maximization and divergence minimization, generative models optimize loss functions to align closely with empirical data distributions. These models excel at capturing intrinsic data relationships, enabling them to represent variability and interpolate effectively in sparsely observed or unobserved regions.

In the context of diabetes management, accurate probability estimates are crucial for assessing risks—such as the likelihood of hypoglycemic events given recent insulin intake, carbohydrate consumption, and prior glucose trends. These predictions often involve high-dimensional conditional distributions where the conditioning set includes multivariate, time-dependent covariates (e.g., insulin doses, meal timing, physical activity, and continuous glucose measurements). Estimating such conditional densities is challenging due to data sparsity and nonlinear dependencies. Generative models provide a powerful framework for modeling these distributions, enabling the generation of synthetic but plausible patient trajectories that support downstream predictive modeling and simulation-based decision support. This study focuses on the generative methodologies applied to density estimation in this domain, highlighting their potential for improving clinical insight and personalized care strategies.

#### Normalizing flows

A widely utilized method for density estimation involves sampling data from a basic reference distribution, followed by the learning of a sequence of invertible transformations to adjust it toward a target distribution. Normalizing flows represent a particularly effective class of tractable density architectures that have demonstrated great success in this approach. They employ a series of invertible transformations to map complex distributions to simpler ones, typically a Gaussian distribution. By applying the change of variables formula, normalizing flows enable explicit density calculation, facilitating both efficient density estimation and sampling. However, since normalizing flows have not been utilized extensively by researchers in diabetes healthcare, they are not discussed in detail in this article.

Evidence from the scientific literature contains some works based on normalizing flows targeted at applications in diabetes healthcare or healthcare in general. These research works include the use of conditional normalizing flows for class balancing of imbalanced tabular biomedical data.^11^ Polanska et al. suggest that normalizing flows offer robust, scalable, and flexible methods for improving Bayesian model comparison, making them highly suitable for complex, high-dimensional healthcare datasets, such as those used in diabetes management applications.^12^

#### Deep autoregressive models

Deep autoregressive models use density estimation by treating the probability of each data point as a product of sequential, conditional probabilities, where each current value in a sequence (e.g., a time series) depends on past values. This approach allows the model to compute each component individually and sequentially, with each part relying on previously estimated components.

Although they have also been used to generate nonsequential data such as images, autoregressive models are preferred for sequential data generation. In diabetes healthcare, autoregressive models have mostly been employed for predicting future blood glucose values.^13^ Apart from blood glucose prediction, autoregressive models can be used to generate a time series of blood glucose data to be utilized for other purposes. A study by Zhou et al. shows the use of autoregressive models for the generation of continuous glucose monitoring (CGM) values for error and drift modeling.^14^ Another study employs autoregressive neural networks for the estimation of the risk of hypo- and hyper-glycemic episodes.^15^ One of the benefits of autoregressive models is that they are somewhat interpretable. This incentivizes researchers to use such models in applications in diabetes healthcare since biomedical applications of AI require a higher level of interpretability. Moreover, these models can be computationally efficient and suitable for real-time applications.

#### Variational autoencoders

**Figure 2:**
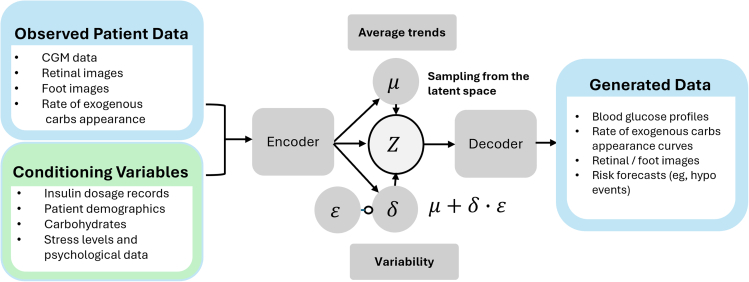
Illustrative architecture of a conditional variational autoencoder designed to generate diabetes-related data as the target output, conditioned on diabetes-specific input features Note that the inputs (observed patient data and conditioning variables) and outputs (generated data) listed here are illustrative examples and do not represent exhaustive lists.

VAEs are approximate density models that target complex data distributions but cannot compute exact probability densities. Instead of providing an exact form of the probability distribution p(x), they introduce latent variables to describe hidden structures or factors in the data and then model the joint distribution over the data and the latent variables. By introducing latent variables, VAEs reduce the computational complexity of modeling high-dimensional data directly, making inference and learning more tractable. Since the true posterior distribution over these latent variables is typically intractable, VAEs utilize variational inference as an approximation method. The encoder encodes two vectors: one for means (μ) and another for standard deviations (σ) of random variables, as shown in Figure 2. Where X is the real data and ϵ is the standard normal distribution. C is the conditional input to condition the generation of data in the conditional variant of the VAE. The decoder uses both the mean and standard deviation during the generative process, mapping real samples to the mean while leveraging the standard deviation to introduce variability, thereby producing distinct outcomes on each iteration.

The dominant data modality in diabetes to be generated using VAEs is images and text. VAEs have been employed to synthesize images of diabetes-related conditions that can be diagnosed using visual data. For example, Sundar et al. propose the use of VAEs for grading abnormalities in diabetes retinopathy images.^16^ Additionally, Li et al. conducted a study using tongue images to diagnose diabetes, proposing a VAE-based classification model.^17^ There are also research studies focused on the prediction and detection of different determinants of diabetes. For example, Lim et al. have used a VAE-based architecture for diverse tasks including glucose forecasting, event detection, and temporal clustering.^18^ Furthermore, a study from Nature Biotechnology demonstrates the use of VAEs for the discovery of drug omics associations in type 2 diabetes.^19^

Some researchers do not use VAEs directly for prediction or detection but instead use them to extract features from the training data before subjecting the features to another deep-learning model for the required task. One such work by Sundas et al. shows the use of VAEs for essential retinal image features from fundus images before providing them to a graph convolutional network for classification.^20^ In addition, researchers have used VAEs to augment imbalanced datasets in diabetes healthcare, facilitating their use in machine learning-based tasks.^21^

Furthermore, VAEs have also been utilized to generate electronic health records (EHRs) for patients suffering from different diseases.^22^ Research work from Liao et al. demonstrates the use of VAE for learning patient static information, including sex, age, race, and diabetes status from EHRs time series.^23^

The generative nature of VAEs enables them to identify outliers or anomalies that deviate significantly from the learned patterns.^24^ A article by Li et al. proposes the use of a VAE-based model for self-supervised anomaly detection in retinal images.^25^ A study from Li et al. discusses the use of VAEs in detecting anomalies in time series data from wearable devices.^26^

#### Generative adversarial networks

**Figure 3:**
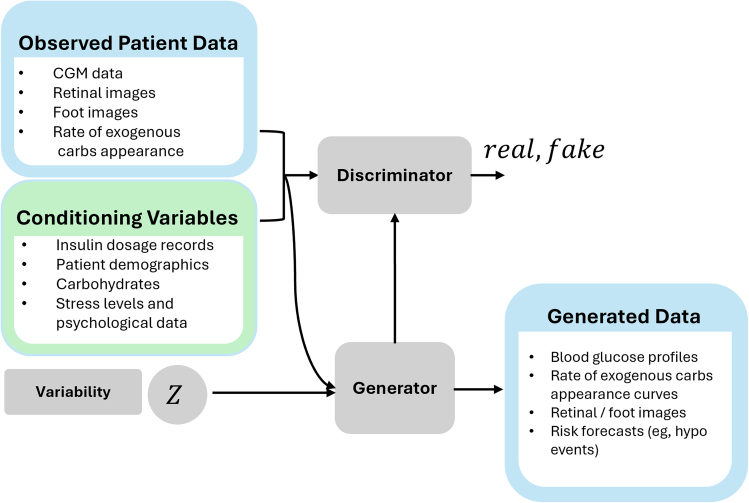
Illustrative architecture of a conditional generative adversarial network designed to generate diabetes-related data as the target output, conditioned on diabetes-specific input features Note that the inputs (observed patient data and conditioning variables) and outputs (generated data) listed here are illustrative examples and do not represent exhaustive lists.

GANs stand out for their unique ability to generate data without explicitly modeling or providing a probability density function (PDF) of the data distribution. GANs are a type of composite model consisting of two types of networks, the discriminator model and the generator model that are trained in an adversarial fashion. Figure 3 shows the schematic of a GAN. In GANs, a generator model creates samples, and a discriminator model tries to distinguish between real data and generated data. The generator improves by trying to fool the discriminator, leading to realistic sample generation. The generator and discriminator models in GANs are deep neural networks and may be set up using different types of layers based on the type of application and data modality. This quality of GANs makes it more flexible and suitable for use in various applications.

The training process of a GAN is commonly framed as a *minimax optimization problem*, where the generator G and discriminator D play adversarial roles. The goal is for D to correctly distinguish real samples from fake ones, while G aims to generate samples that are indistinguishable from real data. This interaction can be formalized through the following minimax objective:

(Equation 1) $$\min_{G}\max_{D}\left\{E_{x∼p_{\text{data}}}\left[\log\;D\left(x\right)\right]+E_{z∼p_{z}}\left[\log\left(1-D\left(G\left(z\right)\right)\right)\right]\right\}$$

This expression defines a two-player game. The discriminator D seeks to maximize the expected log likelihood of assigning high probabilities to real data $x∼p_{\text{data}}$ (first term), and low probabilities to generated samples G(z), where $z∼p_{z}$ is drawn from a prior distribution (second term). Conversely, the generator G attempts to minimize this objective by generating data that maximizes the discriminator’s error, effectively *fooling* D.

Though GAN was proposed in 2014 by Goodfellow et al.^27^ and has predominantly been used for image-generation tasks, researchers in diabetes healthcare technology have employed it for several applications over the past five years. In diabetes healthcare, GANs have been used in blood glucose time series generation to predict future values or to augment datasets. One such study from Zhu et al. proposes GluGAN, which generates time series data in the future.^28^ Noguer et al. propose a GAN-based method to generate personalized blood glucose data.^29^ Moreover, a study from Seo et al. also presents a GAN-based augmentation strategy to enhance the performance of hypoglycemia classifiers.^30^

Cichosz et al. presented the conditional generation of blood glucose data conditioned on HbA1C values using a conditional GAN (CGAN).^31^ Mujahid et al. presented the conditional generation of blood glucose values using insulin as the conditional input.^32^ This work signifies the capability of CGANs to accurately approximate the relationship existing between insulin and blood glucose. Following this study, a type 1 diabetes simulator was proposed using a sequence-to-sequence CGAN model, with insulin and carbohydrates as inputs to generate blood glucose values.^6^ A article from Nouger et al. demonstrates the modeling of the rate of exogenous glucose appearance (RA) using a CGAN.^33^ A article from Kalita et al. proposes an adaptive CGAN architecture that can be used to augment diabetes-related data.^34^ A study from Nair et al. proposed the use of GAN for modeling clinical biomarker profiles.^35^ GANs have also been employed to synthesize realistic EHRs. Baowaly et al. developed a GAN-based architecture to synthesize realistic EHRs.^36^ Another work from Bernardini et al. presented the use of CGAN for the imputation of missing data in multi-diabetic centers’ routine EHR data.^37^

Apart from time series and text data, GANs have been used to generate image-based data in diabetes. A article from Wang et al. discusses the use of GAN for the diagnosis of diabetic retinopathy from images, solving the problem of the lack of labeled data in diabetes retinopathy.^38^ Zhou et al. propose DR-GAN for the synthesis of high-resolution fundus images that can be altered to be used in a diabetic retinopathy grading and lesion segmentation model.^39^ Research work from Jishnu et al. employed a CGAN for the segmentation of foot ulcers.^40^ Bloch et al. used a CGAN in a pix2pix configuration for the synthesis of images in Infection and Ischemia classification in diabetic foot ulcers.^41^

#### Transformer

**Figure 4:**
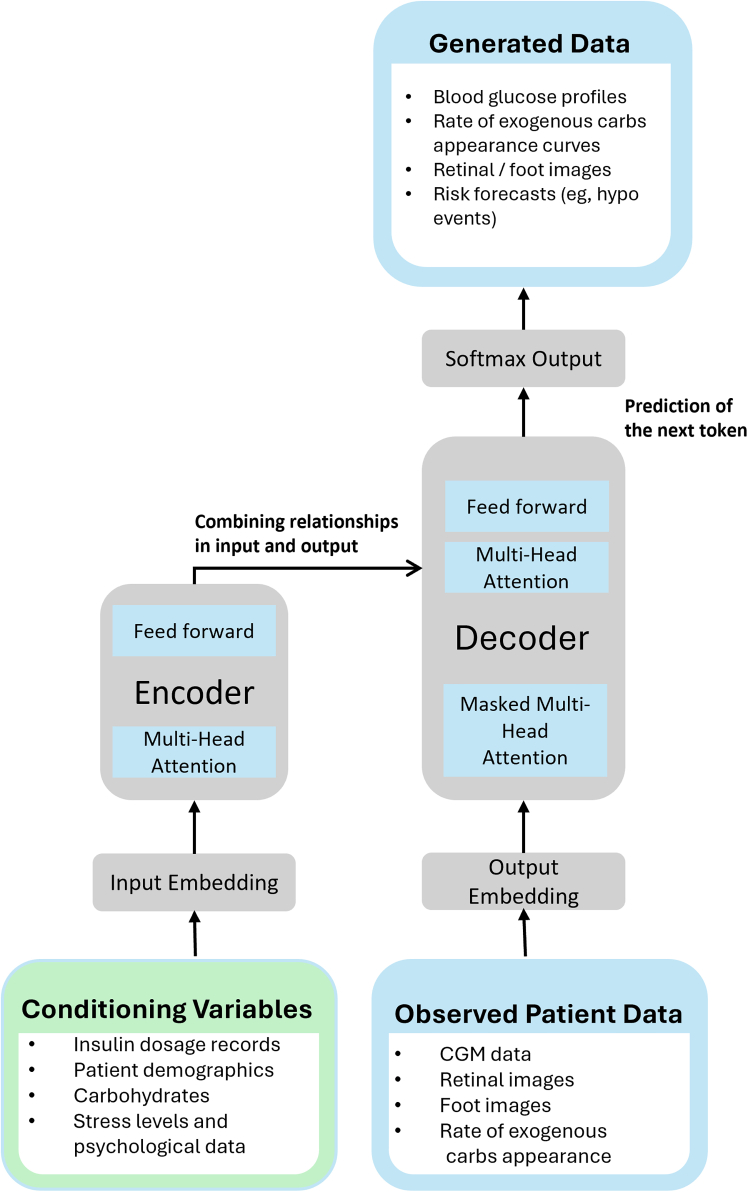
Illustrative architecture of a transformer model adapted as a conditional generative framework for generating diabetes-related data, conditioned on diabetes-specific input features Note that the inputs (observed patient data and conditioning variables) and outputs (generated data) listed here are illustrative examples and do not represent exhaustive lists.

Transformers leverage density estimation by modeling the joint probability of a sequence using self-attention mechanisms.^42^ This approach captures dependencies among all elements in the sequence simultaneously, assigning attention weights to estimate their contributions to the overall probability. Transformers are central to modern LLMs such as chat generative pre-trained transformer (ChatGPT), driving much of the recent advancements in generative AI.^43^ Figure 4 illustrates the general transformer architecture, consisting of encoder and decoder components. Transformers have demonstrated exceptional performance in language and image generation tasks. In generative configurations, they can be used autoregressively or integrated into VAE or GAN architectures. Their ability to process entire sequences in parallel allows for efficient task execution.

In diabetes healthcare, the transformer model has been adopted for several applications. Apart from its use in LLM-based applications, the transformer has majorly been used in image-based applications such as image classification, generation, and reconstruction applications in diabetes.^44^ The applications are mostly targeted at diabetic retinopathy or diabetic neuropathy, with a plethora of research articles being published in these areas. However, not all of these applications are generative. These research works often use the transformer as a classification model for the prediction/classification of image/time series data.

Researchers have also utilized transformer-based LLMs such as ChatGPT for various tasks in diabetes healthcare. The use of transformers to develop chatbots in diabetes healthcare has been explored by researchers.^45^ Dao et al. proposed a multi-modal digital solution based on a transformer-based LLM focused on the active prevention of diabetes.^46^ Healey et al. proposed that transformer-based LLMs can produce accurate and safe summaries of CGM time series data.^47^ Sharma et al. explored the use of transformer-based LLM as a substitute for diabetes educators.^48^ A article from Jeong et al. showed the surprising capability of LLMs to select the most suitable features in a diabetes classification model.^49^ In their work, they provided the LLM with the names of the features and the predictive task, and the LLM returned the most relevant features for that task. Cappon et al. demonstrated the use of a GPT-based LLM along with a digital twin model to provide explanations and contextual information about the recommended therapy.^50^ Sophia Meywirth proposed an LLM-based methodology for a lifestyle behavior change coach.^51^ In this article, the author argues in favor of LLM-based coaches instead of emails or text messaging.

#### Diffusion models

Diffusion models are generative models that create data by simulating a gradual noise-removal process. They involve a forward process that systematically adds Gaussian noise to the data in T steps, transforming it into pure noise. The model then learns a reverse process to gradually denoise this noise back to the original data.

**Figure 5:**
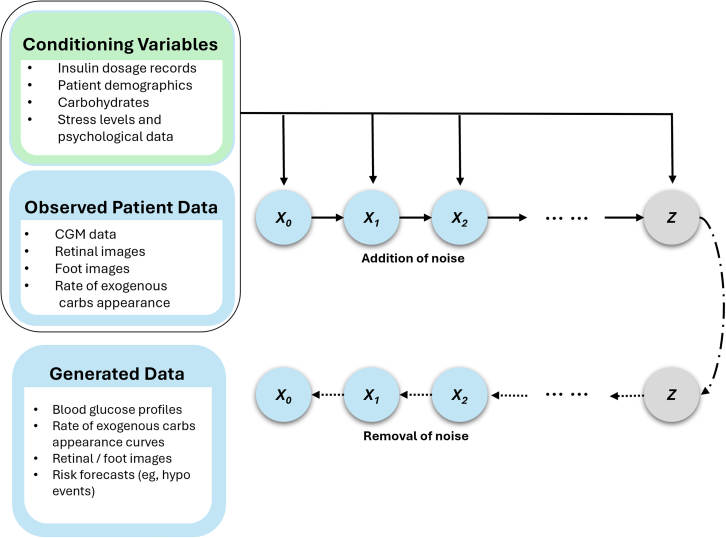
Illustrative architecture of a diffusion model designed for the conditional generation of diabetes-related data, utilizing diabetes-specific input features to guide the progressive refinement of the generated output Note that the inputs (observed patient data and conditioning variables) and outputs (generated data) listed here are illustrative examples and do not represent exhaustive lists.

By training to minimize the discrepancy between the forward and reverse processes, diffusion models can generate realistic data, often achieving high-quality results. Figure 5 shows the general block diagram of a diffusion process.

Diffusion models are versatile and can be adapted across multiple data modalities, including images, text, audio, and time series data. Moreover, like all the other architectures discussed, diffusion models are capable of generating data conditioned on other inputs. This flexibility makes them suitable for a wide range of applications. In healthcare, particularly in diabetes healthcare, diffusion models hold promise for generating synthetic patient data to enhance predictive models and support personalized medicine. However, the current literature shows limited evidence of the use of diffusion models in diabetes. Their use in diabetes healthcare has mostly been focused on image synthesis tasks. They have been used to generate retinal or diabetic foot images for dataset augmentation or image grading.^52^^,^^53^ Kotelnikov et al. have demonstrated the use of diffusion models to model tabular data, including tabular diabetes datasets.^54^ Dong et al. have presented an approach based on diffusion models for generating high-resolution magnetic resonance spectroscopic images (MRSI) from low-resolution MRSI images.^55^ MRSI plays an important role in understanding diseases such as diabetes and cancer.

## Insights

In the following discussion, the authors share insights on specific aspects of generative AI as it applies to diabetes management, including its clinical potential, ethical implications, technical limitations, and the need for multidisciplinary collaboration in diabetes management, reflecting current research and future recommendations for safe, equitable AI use.

### Data-related challenges and model selection

While generative AI has demonstrated remarkable versatility across diabetes healthcare applications, we strongly advocate for a data modality-driven approach in model selection. Our review reveals that generative AI’s success in diabetes applications stems not just from its ability to mimic diverse data types but crucially from the careful alignment between model architectures and data characteristics. We assert that the effectiveness of generative models in diabetes healthcare is fundamentally tied to how well their architecture matches the intrinsic properties of the target data modality. Models such as recurrent neural networks or long short-term memory excel in capturing temporal dependencies in CGM data, while CNNs and GANs suit retinal imaging and wound analysis. Autoregressive and transformer-based models work best for clinical notes, and GANs or VAEs handle time-series and tabular data effectively. Despite the absence of universal standards for model selection, prioritizing data modality-driven approaches over popular architectures enhances the reliability and effectiveness of generative AI in diabetes applications, benefiting both research and clinical practice.

The success of generative AI in diabetes healthcare depends on addressing data quantity and completeness. Limited data availability and missing values, common in healthcare, must inform model selection to avoid overfitting and ensure robustness. Some architectures handle missing data better, making them preferable for incomplete datasets. In clinical settings, where missing values are often unavoidable, researchers should adopt a two-pronged approach: evaluate models based on data volume and prioritize those robust to missing values during preprocessing and selection. This ensures more reliable and effective generative AI applications in real-world diabetes healthcare.

Deep generative models face significant training instabilities. GANs are notoriously difficult to stabilize, suffering from issues such as mode collapse, oscillations, and sensitivity to hyperparameters. VAEs require a careful balance between reconstruction loss and Kullback–Leibler divergence to avoid overfitting or latent space collapse, including posterior collapse, which renders latent variables ineffective. Autoregressive models struggle with vanishing gradients in long sequences and slow, sequential training, limiting their efficiency. These challenges hinder the application of generative AI in diabetes healthcare. Ensuring stable training and reliable outputs is essential to leverage their potential fully. The inherent complexity of physiological processes being modeled should be the primary driver in determining data requirements for generative AI in diabetes healthcare. While simpler, well-defined processes, such as RA curves, may require modest datasets, complex phenomena such as 24-h blood glucose profiles in type 1 diabetes demand substantially larger and more diverse datasets due to their multifactorial nature. These profiles are influenced by numerous dynamic factors, including insulin administration, physical activity, meal intake, and psychological stress, each adding layers of variability that must be captured. Therefore, we advocate for a nuanced, complexity-driven approach to dataset requirements in diabetes-focused generative AI, where data volume requirements are calibrated to the physiological complexity being modeled, ensuring both efficient resource utilization and model reliability across different applications. Recent advances in generative AI emphasize the importance of structure-aware and relational modeling, particularly in high-stakes domains such as diabetes care. Physiological processes such as glucose-insulin dynamics are governed by complex, causal relationships rather than simple correlations. Incorporating causality into deep generative models (DGMs) enhances their ability to simulate not only plausible data but also meaningful cause-effect interactions—crucial for applications such as intervention simulation and digital twins. This can be achieved by enforcing temporal ordering, integrating structural causal models, or training on interventional and counterfactual data. In this context, graph-based techniques such as Graph Neural Networks (GNNs) provide powerful tools for representing and reasoning over structured dependencies in healthcare data. For example, GNNs have been used to model contact tracing and optimize epidemic interventions^56^ and to analyze heterogeneous healthcare data during the COVID-19 pandemic.^57^ While GNNs are not generative models per se, they can be integrated into generative pipelines to encode causal graphs, model patient similarity networks, or simulate disease progression pathways. Applying similar principles to diabetes care can enable the development of more robust and personalized digital twins, where GNNs help capture patient-specific physiological networks or treatment-response graphs within the generative modeling framework.

### Pretraining in generative models

Pretraining has become a pivotal strategy in the development of generative AI models for healthcare applications. While often associated with transformers and LLMs, pretraining is also applicable to other generative models such as VAEs, GANs, and diffusion models. By first learning generalizable representations from large, unlabeled data, generative models can be effectively fine-tuned on small, context-specific datasets, improving robustness and accelerating convergence. In diabetes healthcare, where labeled data can be limited and expensive to obtain, pretraining can dramatically reduce the burden of manual annotation and improve performance in downstream tasks such as personalized modeling, synthetic data generation, glucose prediction, and anomaly detection. This approach addresses the challenge of limited labeled data in healthcare and has been shown to improve both generalization and predictive performance. A recent survey consolidates the evidence that pretraining methods, including contrastive learning, masked modeling, and transfer learning, are becoming essential for building robust generative AI systems in healthcare.^58^ These advancements underscore the growing role of pretraining in enabling generalizable and data-efficient generative AI for diabetes and broader medical applications.

### Digital twins and likelihood estimation

Building on early efforts such as the Archimedes model, which demonstrated the value of computational simulations in diabetes care,^59^ the concept of digital twins has emerged as a promising advancement, shifting from static simulations to interactive, personalized models of patient physiology. Generative AI’s greatest potential in diabetes healthcare lies in evolving from static models to interactive digital twins that simulate personal physiological responses. Properly trained with causal relationships, deep generative models can approximate complex systems such as glucose-insulin dynamics. We propose using these models to create personalized simulation environments for testing therapeutic interventions, shifting from predictive tools to interactive platforms. Connected to real-time patient data from wearable devices, these digital twins could help patients explore glycemic trends under various scenarios, fostering experimentation and learning. Prioritizing this approach would transform diabetes management from reactive to proactive, enabling the virtual testing of interventions and bridging theoretical models with personalized care.

Generative AI’s probability density estimation capabilities should be utilized as a key safety mechanism in diabetes healthcare. Beyond data generation, these models can estimate sample likelihoods to guard against malicious attacks and unintentional errors. We propose a dual-purpose use: defending against adversarial attacks in medical imaging, where subtle manipulations risk misdiagnoses, and validating simulation environments to ensure physiological plausibility. Incorporating likelihood-based validation as a standard safety feature would enable the automatic rejection of unrealistic inputs in simulations and protect diagnostic systems from adversarial threats. This approach adds a vital layer of safety, ensuring decisions and experiments align with physiological and clinical realities.

### Ethical, transparent, and reliable generative artificial intelligence

Generative AI offers transformative potential in diabetes healthcare but requires robust validation frameworks and ethical guidelines. The benefits of synthetic data must be balanced with quality control, transparency, and clinical reliability, as AI-driven decisions directly impact patient outcomes. The lack of standardized evaluation frameworks hinders safe deployment, highlighting the need for metrics that connect statistical performance with clinical relevance, especially for underrepresented populations. We propose advancing generative AI by developing validation methodologies for synthetic data, establishing ethical standards for transparency and privacy, and creating metrics that address both technical and clinical needs. This approach builds trust and ensures responsible implementation, particularly benefiting underserved populations.

Explainability is crucial for applying generative AI in diabetes healthcare, where trust and accuracy are vital. Despite its capabilities, generative AI’s opacity poses challenges in validating outputs. Balancing performance with interpretability is essential, as healthcare providers and patients need clarity on AI-assisted insights. Latent variable DGMs such as VAEs provide low dimensional data representations that are difficult to interpret. GANs excel at generating realistic data but lack transparency due to their adversarial design, and autoregressive models obscure the impact of earlier decisions. Advancing interpretable DGMs through techniques such as disentangled representations, feature attribution, and latent space visualization can address these issues. Hybrid models combining DGMs with interpretable approaches, such as probabilistic models or decision trees, offer a promising balance between performance and explainability, meeting both technical and ethical demands in medical AI.

The adoption of generative AI in diabetes healthcare must comply with regulatory frameworks such as the European AI Act and the FDA’s Good Machine Learning Practices.^60^^,^^61^ These frameworks classify healthcare AI as high-risk due to its impact on clinical decisions, emphasizing transparency, accountability, and safety. By requiring robust validation, explainability, and bias mitigation, these regulations address key challenges in deploying generative AI. Generative models, particularly those producing synthetic data, highlight both the potential and risks of this technology. To meet regulatory standards and unlock their promise, systems must integrate rigorous validation processes and prioritize ethical considerations.

### The role of large language models

LLMs have the potential to revolutionize diabetes healthcare by centralizing knowledge and managing diverse data modalities to generate meaningful insights. Their ability to unify disparate data streams into interpretable outcomes makes them invaluable in patient-centric systems, enhancing engagement and clinical outcomes. Startups such as Lark Health and SNAQ leverage LLMs for virtual coaching, real-time data analysis, dietary management, and simplifying complex models for patients and caregivers. While advocating for LLM integration in diabetes-focused digital health, we emphasize the need for ethical and practical safeguards. Unregulated deployment risks misuse of sensitive data and context-free recommendations, with many developers prioritizing technical achievements over patient safety, underscoring the importance of ethical responsibility in medical applications.

LLMs pose significant risks if misused, potentially providing harmful and misleading health advice without medical expertise. For instance, they could misinterpret CGM data and suggest incorrect insulin dosages or lifestyle changes, endangering patient health. The ease of deploying AI-powered health tools allows unqualified individuals or organizations to create seemingly credible systems, undermining professional judgment and promoting misinformation. This can discourage patients from consulting professionals or following established treatment plans. To address these risks, strict regulatory frameworks, certification processes, and validation protocols are essential. While LLMs have immense potential to enhance diabetes care, their use must prioritize ethics and reliability.

### Qualitative comparative evaluation of generative models

Beyond modality-driven alignment, selecting the right generative model also requires understanding its relative trade-offs. VAEs offer stable training and smooth latent spaces, making them suitable for time-series and EHR data, though outputs may lack fine detail. GANs produce highly realistic samples and support tasks such as retinal image generation or CGM data synthesis, but suffer from training instability and mode collapse. Diffusion models, a newer alternative, offer diverse, high-fidelity outputs with more stable training and better mode coverage but demand heavy computation and slow sampling. Transformers perform well in long-range glucose prediction, text generation, and multimodal fusion, benefiting from pretraining, though they require substantial data and compute, and may hallucinate implausible outputs. Overall, VAEs excel in representation learning and anomaly detection, GANs in photorealistic synthesis, diffusion models in diverse image generation with fewer artifacts, and transformers in sequence prediction and clinical language tasks. Each model’s utility in diabetes applications depends on the task, data availability, and trade-offs between fidelity, stability, and scalability. A detailed comparative summary of these models and their diabetes-related use cases is provided in Table S2.

## Conclusion

This article reviews the use of generative AI in diabetes healthcare, emphasizing its ability to approximate underlying probability distributions and generate realistic data for scarce domains. These models accommodate various data modalities in diabetes healthcare, enabling applications such as anomaly detection, predictive modeling, and digital twins. Yet, challenges remain, including data scarcity, interpretability, and training instability. Overcoming these obstacles will enhance the clinical utility of generative AI, improving diagnostic accuracy, therapies, and patient outcomes. Future work should focus on strengthening model robustness, explainability, validation, and addressing ethical considerations in clinical use.

## Acknowledgments

This work was partially supported by the grant PID2023-149837OB-I00 funded by MCIN/AEI/10.13039/501100011033, and by the Government of Catalonia under grants 2017SGR1551 and 2024-PROD-00051.

## Author contributions

J.V.: conceptualization, investigation, data analysis, visualization, writing – original draft, writing – review and editing, funding acquisition; O.M.: investigation, data analysis, writing - original draft, writing - review and editing, visualization, project administration; I.C.: data analysis, writing – original draft, writing - review and editing, visualization, supervision; A.B.: writing - review and editing, data analysis, project coordination.

## Declaration of interests

The authors declare no competing interests.

## Supplemental information

Document S1. Figure S1 and Table S2

Table S1. Overview of published studies using deep generative models in diabetes research
