# Supplementary material for: Generative artificial intelligence in diabetes healthcare
Source: iScience. 2025 Jul 5;28(8):113051. doi: 10.1016/j.isci.2025.113051 (PMC12284359; doi:10.1016/j.isci.2025.113051)
Supplement: Document S1. Figure S1 and Table S2 [file mmc1.pdf]

iScience, Volume *28*

Supplemental information

Generative artificial intelligence in diabetes healthcare

Josep Vehi, Omer Mujahid, Aleix Beneyto, and Ivan Contreras

Table S2: Qualitative comparison of generative model types in diabetes healthcare

Model Type	Typical Tasks	Data Types	Strengths	Weaknesses	Diabetes Use Cases
**VAE**	Synthetic data generation, anomaly detection, latent encoding	Tabular, time-series, images	Stable training, smooth latent space, works with smaller datasets	Blurry outputs, posterior collapse, less accurate for discrete variables	EHR synthesis, CGM anomaly detection, biomarker modeling
**GAN**	Image/time-series synthesis, data augmentation, semi-supervised learning	Images, tabular, time-series	High realism, supports rare case generation, versatile	Mode collapse, unstable training, large data requirements	Retinal image synthesis, CGM signal generation, DR classification, RA curve generation, modeling glucose-insulin-CHO dynamics
**Diffusion**	High-fidelity image synthesis, dataset balancing, refinement	Medical imaging (retina, OCT), emerging in time-series	Excellent realism, better mode coverage, stable convergence	Slow sampling, high compute demand, newer in practice	DR image generation, augmentation for fairness, training medical staff
**Transformer**	Glucose forecasting, synthetic EHR/text, multimodal modeling	Time-series, text, images	Long-term pattern capture, flexible, leverages pretraining	Resource-heavy, hallucinations, overfitting risk on small data	Glucose prediction, ViT-based DR detection, GPT-style record generation

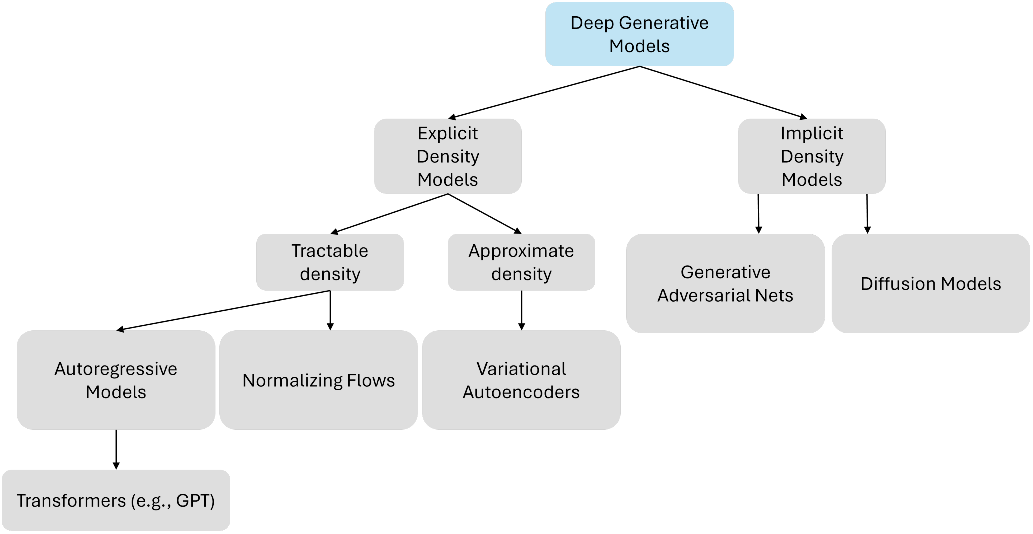

Figure S1: Taxonomy of deep generative models employed in diabetes healthcare
